# Variation and association of leaf traits for desert plants in the arid area, northwest China

**DOI:** 10.1002/ece3.9946

**Published:** 2023-03-24

**Authors:** Hongyong Wang, Jie Yang, Tingting Xie, Li Ma, Furong Niu, Cai He, Lishan Shan

**Affiliations:** ^1^ College of Forestry Gansu Agricultural University Lanzhou China; ^2^ Pingliang institute of soil and water conservation Science Pingliang China; ^3^ Wuwei Academy of Forestry Wuwei China

**Keywords:** desert area, desert plant life forms, leaf economic spectrum, leaf traits, leaf traits trade‐offs

## Abstract

Characterizing variation and association of plant traits is critical for understanding plant adaptation strategies and community assembly mechanisms. However, little is known about the leaf trait variations of desert plants and their association with different life forms. We used principal component analysis, Pearson's correlation, phylogenetic independent contrasts, linear mixed model, and variance decomposition to explore the variation and association of 10 leaf traits in 22 desert plants in the arid area of northwest China. We found that: (1) the contribution of interspecific variation to the overall variation was greater than the intraspecific variation of all the studied leaf traits; (2) intraspecific and interspecific variation in leaf traits differed among life forms. Some leaf traits, such as tissue density of shrubs and specific leaf area of herbs, exhibited greater intraspecific than interspecific variation, while other traits exhibited the inverse; (3) desert shrubs corroborate the leaf economic spectrum hypothesis and had a fast acquisitive resource strategy, but herbs may not conform to this hypothesis; (4) there were trade‐offs between leaf traits, which were mediated by phylogeny. Overall, our results suggest that interspecific variation of leaf traits significantly contributes to the total leaf traits variation in desert plants. However, intraspecific variation should not be overlooked. There are contrasts in the resource acquisition strategies between plants life forms. Our results support understanding of the mechanisms underlying community assembly in arid regions and suggest that future works may focus on the variation and association of plant traits at both intra‐ and interspecific scales.

## Graphical abstract

Our results suggest that interspecific variation of leaf traits greatly contributes to the total leaf traits variation in desert plants but intraspecific variation should not be overlooked. There are differences in the resource strategies of plants of different life forms.

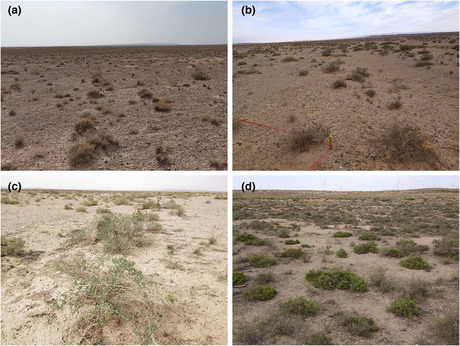

## 1 INTRODUCTION

Plant traits are phenological, physiological, or morphological adaptive and result from long‐term interactions between plants and their environment. These traits can well represent the adaptations of plants to the environmental conditions they inhabit (Violle et al., 2007). Variability and correlations between the traits of plant organs (e.g., leaves) have been well‐documented. Moreover, evolutionary context and environmental changes drive trait variation and determine plant growth responses through trait trade‐offs (Osnas et al., 2013; Reich et al., 2003). The leaf usually has the largest surface area of all the plant organs, giving it the greatest contact with the external environment (Valladares et al., 2000). Additionally, the leaf is closely associated with plant resource utilization ability and can reflect the survival responses of plants when facing adverse conditions (Messier et al., 2016). Therefore, studying the variation and association of leaf traits explains the adaptation strategies of plant individuals to the external environment and gives insight for understanding the community structure and biodiversity maintenance mechanisms (Adler et al., 2013; Fajardo & Siefert, 2016; McGill et al., 2006).

Leaf traits are typical feasible to measure and are often used to compare plant physiological function within and among species. Numerous studies have been conducted to investigate the leaf trait variability, both intraspecific and interspecific variation (Kichenin et al., 2013; Messier et al., 2016; Wright et al., 2004). Morphological, anatomical, chemical elements, and physiological characteristics of leaves from the same species can make adaptive adjustments with the environmental changes (Lavorel & Garnier, 2002; Suding et al., 2008). There are also significant differences between leaf traits among species (i.e., interspecific variation), indicating differences in growth and survival strategies (Navarro & Hidalgo‐Triana, 2021). However, these results are usually based on an assumption that leaf trait variation is much more considerable between species than within species (Garnier et al., 2001; McGill et al., 2006; Reich et al., 1999). Therefore, plant species can be represented by the mean values of a set of their leaf functional traits (Shipley et al., 2016). Contrarily, Hulshof and Swenson (2010) partitioned variation in four leaf traits from a tropical forest in Costa Rica. Results showed intraspecific variation ranged from 36% to 83% of total trait variation and may play an important role in species coexistence and community establishment (Courbaud et al., 2012; Jung et al., 2010). Hence, understanding interspecies and intraspecies variation is crucial to revealing community structure and mechanisms of biodiversity maintenance (Kichenin et al., 2013). This is particularly useful in communities with low species richness since the relative amount of intraspecific variation decreases with increasing species richness (Siefert et al., 2015).

Plants reflect their physiological and morphological adaptions to environments, and plants of the same species likely have a similar environmental adaptive capacity (Díaz & Cabido, 1997). Studies on plant life form leaf traits can reveal the relationship between plants and environmental change at varied spatial scales (Díaz & Cabido, 1997). A study of five Mediterranean shrublands in the most arid region of the Iberian Peninsula reported that 20%–60% of the variation in specific leaf area was attributed to life form, while only 38%–48% of the variation was attributed to environmental drivers (Navarro & Hidalgo‐Triana, 2021). This suggests that the species impact on leaf traits may be greater than that of environmental factors.

The correlations among plant traits reflect the trade‐offs between individuals, species, and communities (Donovan et al., 2011; Navarro & Hidalgo‐Triana, 2021). They also reveal the coping strategy formed by plants in changing environment, which further controls species coexistence (Westoby et al., 2002). The leaf economic spectrum (LES) combines interrelated leaf functional traits to capture plant resource trade‐off strategies globally (Chen et al., 2021; Wright et al., 2004). Fast‐growing acquisition strategy species are at one end of the spectrum, while slow‐growing conservative strategy species are at the other (Reich, 2014; Wright et al., 2004). The total resources available to plants are limited, and plants will often invest more in one trait and consequently invest less in others (Stearns, 1992). Abrahamson (2007) studied the ecological adaptation strategies of *Serenoa repens* and *Sabal etonia* in seasonally dry areas of Florida, the United States. Results indicated that drought stress led to plant dwarfism and decreased leaf size, number, and photosynthetic ability, but extended leaf longevity. Freschet et al. (2010) documented higher specific leaf weight but lower nitrogen content in aquatic and terrestrial plants with increasing elevation in the subarctic region. In short, plants adjust, transform, or compensate their functions according to the resource conditions of their habitats to balance the survival, growth, and reproduction, which is eventually manifested in their anatomical and physiological traits (Navas et al., 2010; Whitman & Aarssen, 2010).

However, not all leaf traits could be considered core traits (i.e., LES traits) in LES studies (Reich, 2014; Wright et al., 2005). The LES traits must be common in many plants and represent the essential and special ecological functions (Wright et al., 2004). Here, we targeted specific leaf area, leaf nitrogen and phosphorus content, and leaf tissue density. These leaf traits have been reported to be the key leaf functional traits in previous studies in drylands (Kitajima & Poorter, 2010; Osnas et al., 2013; Wright et al., 2004). Specific leaf area, leaf nitrogen content, and leaf phosphorus content represent plants' ability to capture light and CO_2_ and thus productivity. Additionally, leaf tissue density represents plants' resistance to environmental pressures (Kitajima & Poorter, 2010; Osnas et al., 2013; Wright et al., 2004). We aimed to analyze trade‐offs among these key leaf traits to verify whether targeted plants align with the LES theory.

Phylogeny is also one of the essential drivers of trait variation and strongly influences trait association. For instance, Valverde‐Barrantes et al. (2017) demonstrated that phylogenetic structure explains most of the leaf trait variation in more than 600 species. Losos (2008) proposed a hypothesis suggesting that sympatric species should have similar niches and functional traits. For coexisting species in a community, most leaf traits are supposed to be phylogenetically structured (Heberling & Fridley, 2012). As a result, phylogenetic signals must be considered in analyzing leaf trait correlation. In other words, trait association should be analyzed within the context of interspecific phylogenetic information if a plant trait is phylogenetically conservative (Garland et al., 1992).

Desert plants, the primary producers in desert areas, have evolved a series of morphological, physiological characteristics, and growth strategies to adapt to extreme environments in these arid areas (Gong et al., 2020; Zhang et al., 2017). In arid northwest China, shrubs and herbs are the predominant vegetation types and are widely distributed in diverse habitats. Desert grassland, typical desert, and extreme desert are distributed from southeast to northwest in this region (Zhang et al., 2019). This spatial pattern provides an opportunity to investigate the variation and association of leaf traits along an environmental gradient and to validate the LES hypothesis in dryland shrubs and herbs. We hypothesized that (1) interspecific variation in leaf traits of desert plants is greater than intraspecific variation; (2) plants with different life forms have different resource utilization strategies, among which shrubs have a fast resource acquisitive strategy, and herbs have a conservative resource acquisitive strategy; and (3) desert plants adapt to arid habitats through trade‐offs among leaf traits, and the association of leaf traits is influenced by phylogeny.

## 2 MATERIALS AND METHODS

### 2.1 Study area

**FIGURE 1:**
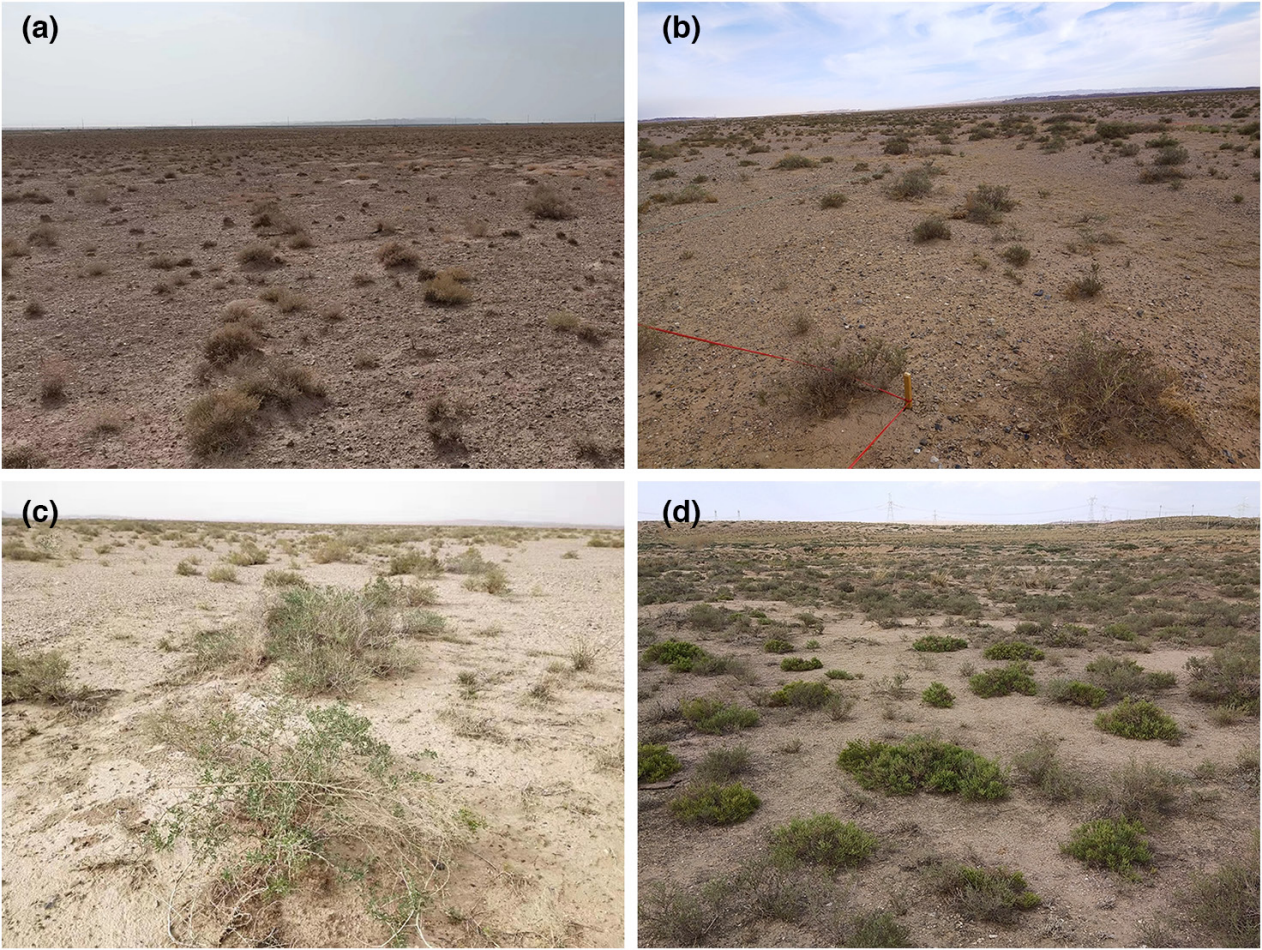
View of the study sites in Jiuquan (a), Zhangye (b), Wuwei (c), and Baiyin (d) in northwest China.

Here, we focused on northwestern China, where desert plants are widely distributed. This area is typically arid/semiarid with sandy soils and the soil organic matter is highly mineralized and the organic matter content is usually low. The climate is a continental monsoon with an average annual rainfall of less than 350 mm, a large temperature difference between day and night, long sunlight hours, and frequent severe weather events such as droughts (Shuai et al., 2022; Wang et al., 2019) (Figure 1).

### 2.2 Sampling design

We set up a sample strip of ca. 900 km in northwest China and selected four sites (i.e., Jiuquan, Zhangye, Wuwei, and Baiyin) for community survey and leaf traits characterization (see Table 1 for details regarding each study site). We selected a shrub/grass community with flat ground and unaffected by grazing in each site. Next, three 50 m × 50 m plots were established and five 10 m × 10 m quadrats were set up at the diagonal ends and midpoints of each plot to conduct the community survey. Species numbers in each quadrat were recorded to further determine targeted species based on criteria of relative abundance >1% (Cheng et al., 2016). There were a total of 10 shrub and 12 herb species targeted (see Table 2 for community survey results).

**TABLE 1:** Geographical and climatic information of the study sites.

Site	Jiuquan	Zhangye	Wuwei	Baiyin
Latitude	39°46′	39°18′	37°63′	36°52′
Longitude	98°23′	100°21′	102°58′	103°42′
Altitude (m)	1500	1590	1775	1947
Annual average temperature (°C)	7.50	6.00	7.80	9.00
Annual average precipitation (mm)	86	131	165	225
Annual average evaporation (mm)	2038	2003	2205	1550
Annual sunshine hours (h)	3228	3075	3000	2580
Habitat type	Extreme desert	Typical desert	Typical desert	Desert grassland

**TABLE 2:** Community survey results from the four study sites in northwest China.

Site	Species	Family	Life form	Species abundance
Jiuquan	*Reaumuria soongarica*	Tamaricaceae	Shrub	116
	*Nitraria tangutorum*	Zygophyllaceae	Shrub	150
	*Ephedra sinica*	Ephedraceae	Shrub	205
	*Kalidium foliatum*	Chenopodiaceae	Shrub	11
	*Asterothamnus alyssoides*	Asteraceae	Shrub	13
	*Salsola passerina*	Amaranthaceae	Shrub	95
	*Sympegma regelii*	Amaranthaceae	Shrub	31
	*Salsola collina*	Amaranthaceae	Herb	12
	*Bassia dasyphylla*	Amaranthaceae	Herb	35
	*Arnebia guttata*	Boraginaceae	Herb	12
	*Zygophyllum gobicum*	Zygophyllaceae	Herb	23
	*Sophora alopecuroides*	Fabaceae	Herb	11
	*Halogeton arachnoideus*	Amaranthaceae	Herb	9
Zhangye	*Reaumuria soongarica*	Tamaricaceae	Shrub	294
	*Nitraria tangutorum*	Zygophyllaceae	Shrub	243
	*Kalidium foliatum*	Amaranthaceae	Shrub	46
	*Anabasis brevifolia*	Amaranthaceae	Shrub	20
	*Salsola passerina*	Amaranthaceae	Shrub	72
	*Salsola collina*	Amaranthaceae	Herb	61
	*Bassia dasyphylla*	Amaranthaceae	Herb	35
	*Arnebia guttata*	Boraginaceae	Herb	16
	*Zygophyllum gobicum*	Zygophyllaceae	Herb	36
	*Sophora alopecuroides*	Fabaceae	Herb	31
	*Halogeton arachnoideus*	Amaranthaceae	Herb	25
	*Eragrostis pilosa*	Poaceae	Herb	29
	*Suaeda glauca*	Amaranthaceae	Herb	15
Wuwei	*Reaumuria soongarica*	Tamaricaceae	Shrub	182
	*Nitraria tangutorum*	Zygophyllaceae	Shrub	49
	*Kalidium foliatum*	Amaranthaceae	Shrub	515
	*Asterothamnus alyssoides*	Asteraceae	Shrub	15
	*Anabasis brevifolia*	Amaranthaceae	Shrub	16
	*Salsola passerina*	Amaranthaceae	Shrub	65
	*Zygophyllum xanthoxylon*	Zygophyllaceae	Shrub	56
	*Zygophyllum gobicum*	Zygophyllaceae	Herb	77
	*Sophora alopecuroides*	Fabaceae	Herb	25
	*Halogeton arachnoideus*	Amaranthaceae	Herb	12
	*Suaeda glauca*	Amaranthaceae	Herb	13
Baiyin	*Reaumuria soongarica*	Tamaricaceae	Shrub	345
	*Nitraria tangutorum*	Zygophyllaceae	Shrub	52
	*Kalidium foliatum*	Amaranthaceae	Shrub	178
	*Asterothamnus alyssoides*	Asteraceae	Shrub	105
	*Salsola passerina*	Amaranthaceae	Shrub	246
	*Zygophyllum xanthoxylon*	Zygophyllaceae	Shrub	33
	*Tamarix chinensis*	Tamaricaceae	Shrub	86
	*Zygophyllum gobicum*	Zygophyllaceae	Herb	53

	*Artemisia frigida*	Asteraceae	Herb	40
	*Achnatherum splendens*	Poaceae	Herb	18
	*Heteropogon contortus*	Poaceae	Herb	68
*Artemisia desertorum*	Asteraceae	Herb	35

A total of 3–10 plants of each targeted species were randomly chosen in each plot, with a total of 9–30 plants per site. From each plant, 5–10 g of healthy mature leaves was collected and kept in a portable refrigerator at −4°C, and then transported to the laboratory for leaf trait analyses.

### 2.3 Leaf traits

Fresh leaf was weighed to derive fresh weight and scanned to determine leaf surface area and leaf volume using WinRHIZO (Regent Instruments Inc., Quebec City, QC, Canada). Leaf samples were then oven‐dried at 65°C for 48 h to determine dry weight. The degree of fleshiness was calculated as fresh leaf weight/dry leaf weight. Leaf water content was calculated as (fresh leaf weight‐dry leaf weight)/fresh leaf weight. Specific leaf area was calculated as leaf surface area/dry leaf weight. Tissue density was calculated as dry leaf weight/leaf volume. Thereafter, the samples were grounded. The leaf carbon and nitrogen content per unit mass were determined using an Element Analyzer (VARIO EL III Element Analyzer, Elementar). The leaf phosphorus content per unit mass was measured using the continuous flow analyzer (San++, Skalar) after H_2_SO_4_–HClO_4_ (4:1, v:v) digestion. All measured traits, their acronyms, ecophysiological functions, and units are provided in Table 3.

**TABLE 3:** Leaf traits measured in this study.

Leaf trait	Acronym	Functions	Unit	References
Degree of fleshiness	DOF	Resource defense	g/g	Chen et al. (2010)
Leaf water content	LWC	Resource capture and defense	%	Chen and Xu (2014)
Specific leaf area	SLA	Resource capture	cm^2^/g	Osnas et al. (2013)
Tissue density	TD	Resource defense	g/cm^3^	Li et al. (2022)
Leaf carbon content	LCC	Resource capture and defense	g/kg	Li et al. (2022)
Leaf nitrogen content	LNC	Resource capture	g/kg	Wright et al. (2004)
Leaf phosphorus content	LPC	Resource capture	g/kg	Wright et al. (2004)
Leaf carbon and nitrogen ratio	LCN	/	/	
Leaf carbon and phosphorus ratio	LCP	/	/	
Leaf nitrogen and phosphorus ratio	LNP	/	/	

### 2.4 Construction of plant phylogeny

The phylogenetic tree was constructed by Phylomatic v3.0, an online software that integrated the skeleton of the phylogenetic tree of Zanne et al. (2014). The software can generate a phylogenetic tree and obtain branch length based on the species checklist according to the Angiosperm Phylogeny Group supertree (APG III; http://www.mobot.org/MOBOT/research/APweb; Webb & Donoghue, 2005; Zanne et al., 2014). The species checklists were first reshaped to the format that can be used in Phylomatic with the R package *plantlist* (Zhang, 2019) statistical platform. Thereafter, the family, genera, and species separated by “/” was copied and pasted to the “taxa” check box of Phylomatic and submitted the input data to Phylomatic. The output is a newick tree that retains the relationship of APG III families and branch length. Lastly, the phylogenetic tree was drawn using the R package *ape* (Paradis et al., 2004).

### 2.5 Data analysis

To compare the magnitude of interspecific or intraspecific variation in leaf traits, we first calculated the interspecific and intraspecific coefficient of variation [i.e., CV = (standard deviation/mean) × 100%] for each leaf trait. We then calculated the contribution of intraspecific and interspecific variation to the total variation for each leaf trait by using a linear mixed model and variance decomposition. We used the R package *lme4* (Bates et al., 2015) AIC function and coefficient of determination (*r* ^2^) to select the optimal linear mixed model. The models include two types: The first one is the model with a random slope and fixed intercept as the equation with 8 degrees of freedom and the second one is the model with a random slope and random intercept as the equation with 16 degrees of freedom. We chose the model with the smallest AIC and the largest *r* ^2^. Among them, interspecific and intraspecific variations were used as fixed factors, and life forms, test sites, the sample of species origin, and species were used as nested random factors. We then used the *glmm.hp* package (Lai et al., 2022) to perform variance decomposition. The ratio between the variance components represents the proportional contribution of each scale change.

In addition, principal component analysis and Pearson's correlation analysis were performed on leaf traits to evaluate the changing patterns between individual traits and resource economy. Pearson's correlation was used to analyze the relationship between leaf traits. Phylogenetic independent contrasts (PIC) were used to analyze the relationship between leaf traits after excluding phylogenetic influence. The PIC was performed with the PIC function of the *ape* package (Paradis et al., 2004). The calculation of the Blomberg's K value and the *p*‐value was performed with the *picante* (Kembel et al., 2010) and *ape* packages (Paradis et al., 2004). All statistical analysis and mapping were completed in R 4.0.3 (R Core Team, 2022) and Origin 2022.

## 3 RESULTS

### 3.1 Total variation of leaf traits

**FIGURE 2:**
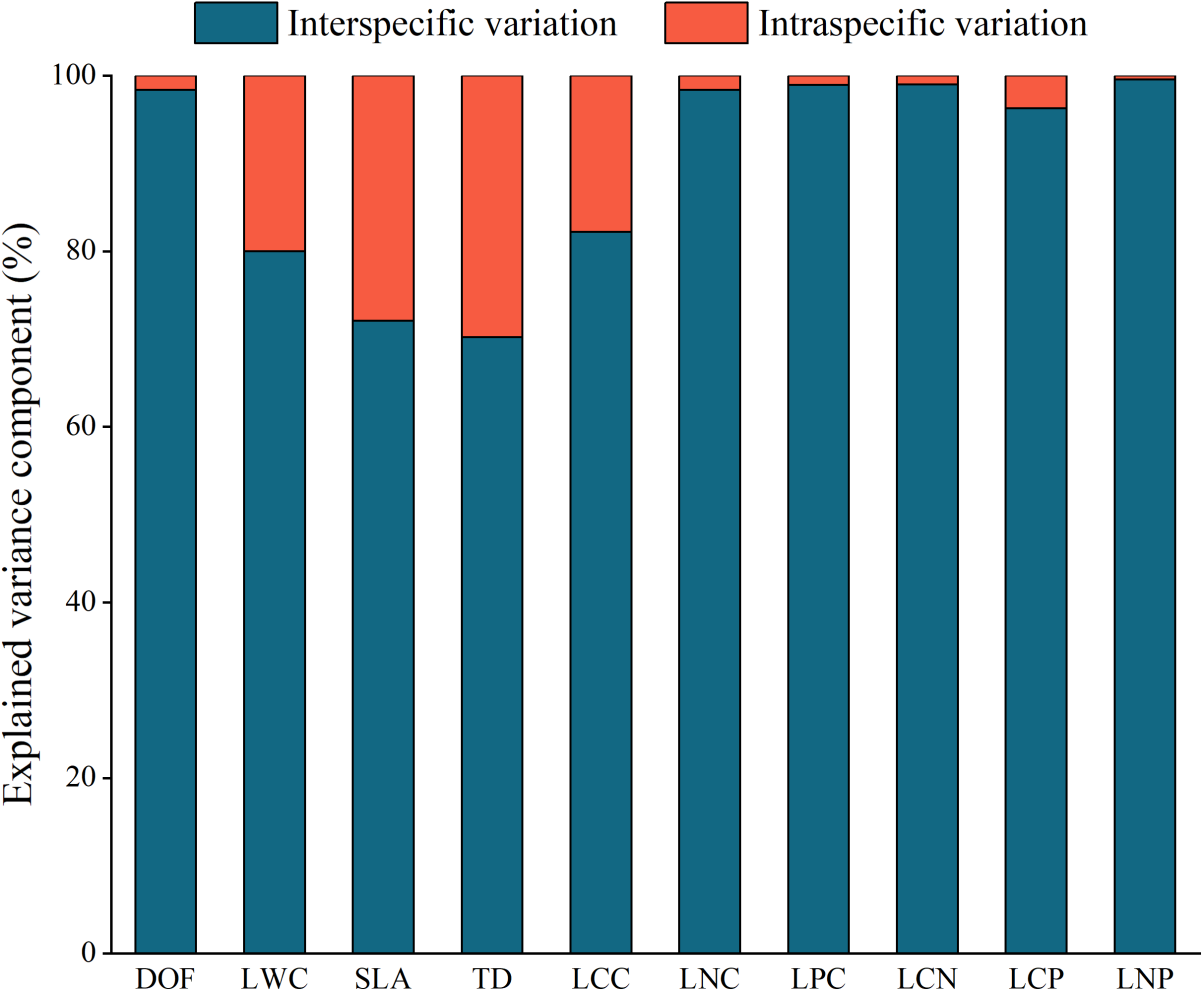
Contribution of interspecific and intraspecific variation to the total variance in measured leaf traits (see Table 3 for acronym definitions).

Interspecific variation in all leaf traits contributed more to total variation than the intraspecific variation (Figure 2). The interspecific contribution in six leaf traits (DOF, LNC, LPC, LCN, LCP, and LNP) was much larger than the intraspecific contribution; the intraspecific contribution was 0.43%–3.7%. The interspecific contribution in four leaf traits (LWC, SLA, TD, and LCC) ranged from 70.22% to 82.18%, and the intraspecific contribution was 17.82%–29.78%.

### 3.2 Intraspecific and interspecific variation in leaf traits within life forms

The leaf traits of desert plants showed different degrees of intra/interspecific variation (Table 4). From the perspective of intraspecific variation, the degree of variation of each leaf trait was relatively low, and the CV was 8.05%–49.97% (Table 4). Leaf water content had minimal intraspecific variation regardless of plant life forms (i.e., shrubs vs. herbs). The intraspecific variation of LCN was the maximum in shrubs, and the intraspecific variation of LCP was the maximum in herbs. Intraspecific variations of DOF, LWC, SLA, TD, and LNC were greater in shrubs than in grasses. LCC, LPC, LCN, LCP, and LNP were greater in herbs than in shrubs.

**TABLE 4:** Leaf traits (mean ± SD) and their coefficients of variation (intraspecific/interspecific) of desert plants with different life forms (shrub vs. herb).

Leaf traits	Shrub	Herb	All
DOF	3.23 ± 1.24	3.26 ± 2.32	3.24 ± 1.62
	(18.13%/94.91%)	(16.90%/62.19%)	(17.55%/54.65%)
LWC	64.45 ± 12.99*	58.95 ± 18.79	62.89 ± 15.05
	(10.90%/18.61%)	(8.05%/31.61%)	(9.14%/25.93%)
SLA	210.43 ± 99.18*	247.66 ± 106.08	220.34 ± 102.31
	(32.35%/35.22%)	(28.75%/22.21%)	(31.09%/30.84%)
TD	0.22 ± 0.14*	0.16 ± 0.09	0.20 ± 0.13
	(44.30%/42.13%)	(33.64%/47.61%)	(38.91%/49.21%)
LCC	33.55 ± 8.37	34.13 ± 10.66	33.84 ± 9.57
	(16.51%/19.34)	(19.96%/20.31%)	(19.84%/19.85%)
LNC	2.08 ± 2.07*	0.70 ± 0.32	1.39 ± 1.63
	(32.64%/122.13%)	(30.16%/33.68%)	(33.15%/119.96%)
LPC	2.68 ± 0.99*	1.96 ± 0.98	2.32 ± 1.05
	(19.36%/31.49%)	(32.06%/41.43%)	(27.79%/37.32%)
LCN	44.64 ± 45.25*	61.35 ± 56.58	52.98 ± 51.83
	(48.51%/54.19%)	(48.57%/42.83%)	(51.58%/47.23%)
LCP	15.75 ± 12.79*	27.47 ± 24.37	21.60 ± 20.29
	(24.71%/53.87%)	(49.97%/62.83%)	(41.07%/68.28%)
LNP	0.76 ± 0.65*	0.60 ± 0.64	0.68 ± 0.65
	(39.69%/90.76%)	(44.84%/80.60%)	(44.63%/83.23%)

*Note*: Numbers before “/” in parentheses indicate intraspecific coefficients of variation, and numbers after for interspecific coefficients of variation. “*” indicates significant differences between shrub and herb (*p* < .05). Abbreviations of leaf traits are given in Table 3.

From the perspective of interspecific variation, the degree of variation of each leaf trait was relatively high, and the CV was from 19.85% to 119.96% (Table 4). For shrubs, the maximum interspecific variation was observed in LNC while the minimum was in LWC. For herbs, the maximum interspecific variation was in LNP, while the LCC had the minimum. There were significant differences in interspecific variation between different life forms. Interspecific variations of DOF, SLA, LNC, LCN, and LNP were larger in shrubs than in herbs, in contrast to greater in herbs than in shrubs in LWC, TD, LCC, LPC, and LCP.

A comparison of the magnitude of intraspecific and interspecific variation revealed that interspecific variation was larger than intraspecific variation for all traits with the exception of TD in shrubs and SLA and LCN in herbs (Table 4). In addition, interspecific variation was larger than intraspecific variation for all leaf traits except for SLA and LCN in all plants pooled (Table 4).

### 3.3 Relationship between leaf traits variation and leaf economic spectrum

**FIGURE 3:**
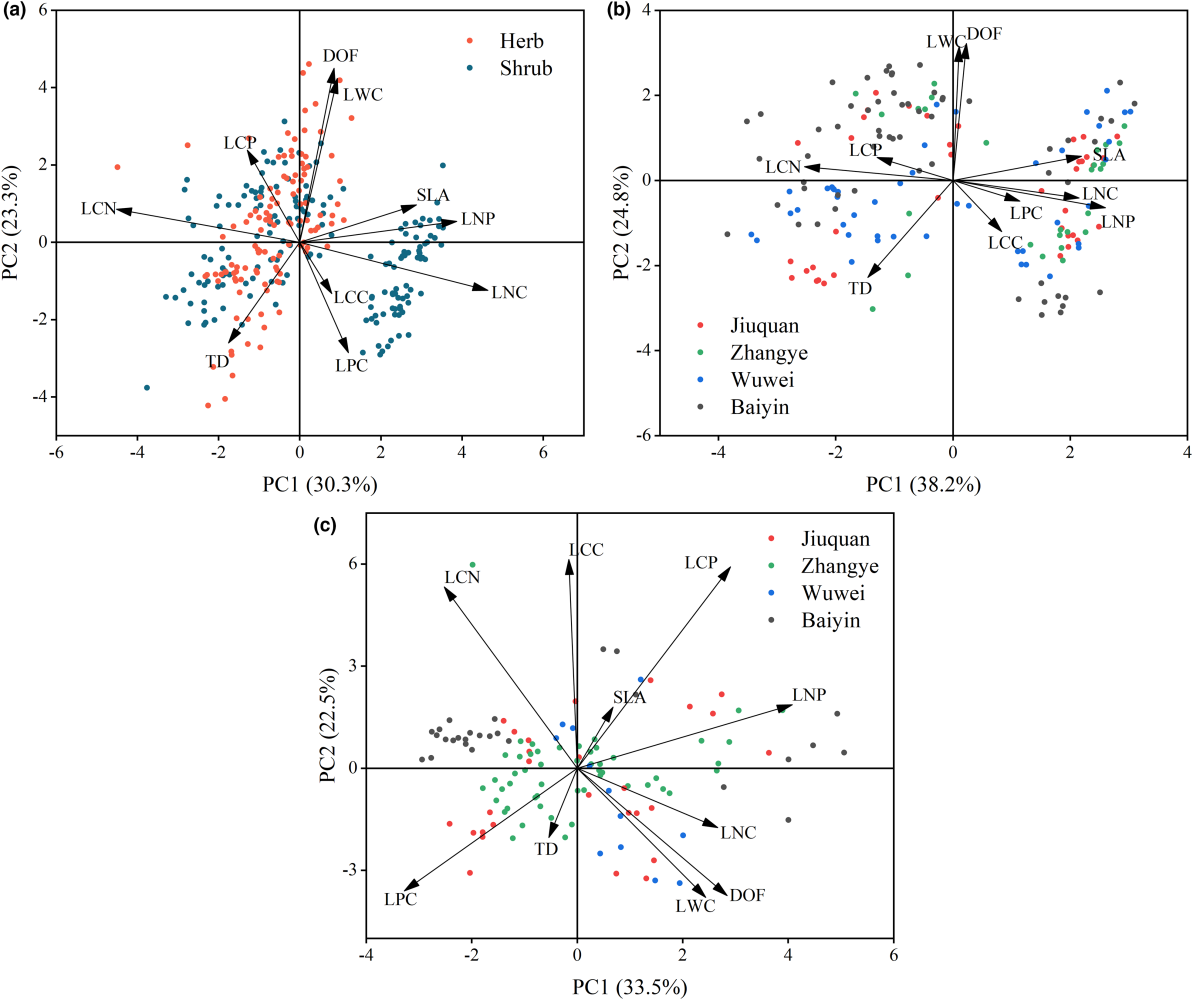
Principal component analysis of leaf traits of all plants (a), shrubs (b), and herbs (c).

Principal component analysis showed that the PC1 and PC2 accounted for 30.3% and 23.3% of the total variations across all plants, respectively (Figure 3a). The PC1 is primarily influenced by SLA, LNC, LCN, and LNP, and the PC2 by DOF, LWC, TD, and LPC. On PC1, shrubs were evenly distributed on both sides, and herbs were mostly distributed on the left side. For leaf traits of shrubs, the PC1 and PC2 accounted for 38.2% and 24.8% of total variations, respectively (Figure 3b). The PC1 is primarily influenced by SLA, LNC, LCN, and LNP, and the PC2 by DOF, LWC, and TD. Shrubs from different study sites were evenly distributed on either side of PC1 or PC2, suggesting that the LES of shrubs were independent of habitat. For leaf traits of herbs, the PC1 and PC2 accounted for 33.5% and 22.5 of the total variations, respectively (Figure 3c). The PC1 is mainly influenced by DOF, LPC, and LNP, and the PC2 by LCC, LCN, and LCP. Similar to the shrubs, the LES of herbs were independent of habitat.

**FIGURE 4:**
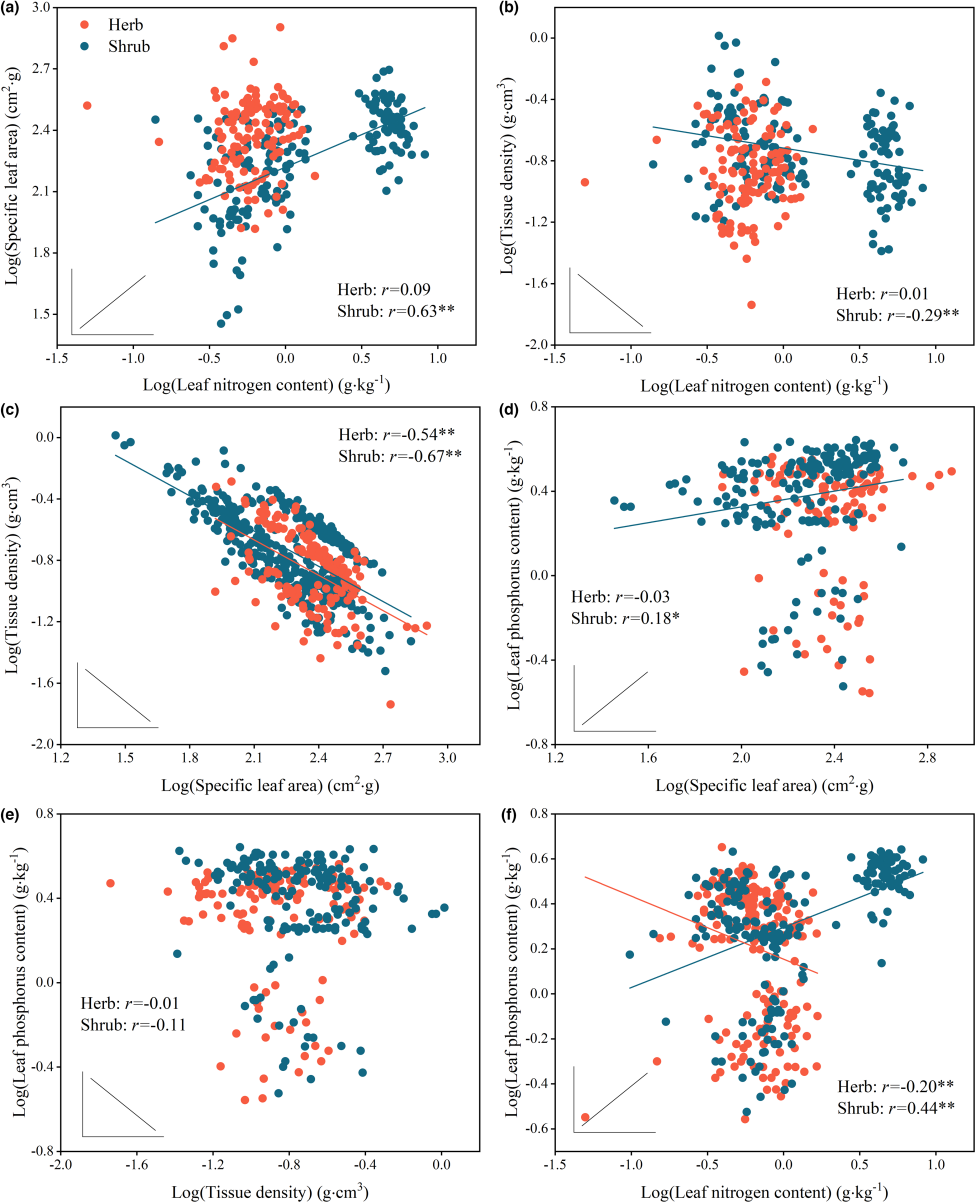
Relationship between LES traits of desert shrubs and herbs. High SLA, LNC, and LPC represent the acquisition resource strategy, and high TD represents the conservative resource strategy. The line in the inset represents the expected relationship between the respective leaf traits according to the LES assumptions. Significant correlations are denoted with asterisks: “*” for *p* < .05, “**” for *p* < .01.

We tested the relationship between LES traits using Pearson's correlation to analyze whether leaf traits of different life forms align with the LES theory. Our results showed that SLA and LNC (Figure 4a, *r* = 0.63, *p* < .01), TD and LNC (Figure 4b, *r* = −0.29, *p* < .01), TD and SLA (Figure 4c, *r* = −0.67, *p* < .01), LPC and SLA (Figure 4d, *r* = 0.18, *p* < .05), and LNC and LPC (Figure 4f, *r* = 0.44, *p* < .01) were correlated in shrubs. TD and SLA (Figure 4c, *r* = −0.54, *p* < .01) and LNC and LPC (Figure 4f, *r* = −0.20, *p* < .01) were correlated in herbs.

### 3.4 Correlations among leaf traits

Pearson's correlation showed correlations among the 10 leaf traits (Table 5, lower‐left diagonal). The morphological traits DOF and LWC, DOF and TD, LWC and TD, and SLA and TD were correlated (Table 5, lower‐left diagonal, *p* < .05). The chemical traits LCC and LNC, LCC and LCP, LNC and LPC, LNC and LCN, LNC and LCP, LNC and LNP, LPC and LCP, LPC and LNP, LCN and LNP, and LCP and LNP were correlated (Table 5, lower‐left diagonal, *p* < .05). There were also correlations between morphological and chemical traits: DOF and LCC, DOF and LPC, LWC and LCC, SLA and LCC, SLA and LNC, SLA and LNP, TD and TD and LCC, TD and LCP (Table 5, lower‐left diagonal, *p* < .05). Phylogenetic analysis showed that only LWC has significant phylogenetic signals among all studied leaf traits (Table 6). In addition, the pairwise correlations of leaf traits determined by the phylogenetic independent contrasts differed from Pearson's correlations (Table 5). The number of trait pairs with significant correlations between morphological traits, chemical traits, and morphological and chemical traits was reduced, implying that the leaf trait variation was related to phylogeny.

**TABLE 5:** Pearson's correlation coefficients for pairwise leaf traits with original data (lower‐left diagonal) and phylogenetic independent contrasts (upper‐right diagonal).

	DOF	LWC	SLA	TD	LCC	LNC	LPC	LCN	LCP	LNP
DOF		0.91**	0.13	−0.29	−0.18	0.17	−0.24	−0.45*	0.17	0.49*
LWC	0.89**		0.03	−0.25	−0.23	0.13	−0.22	−0.39	0.14	0.42
SLA	0.10	0.05		−0.62**	0.59**	0.36	−0.42	−0.12	0.43	0.57**
TD	−0.40 **	−0.33**	−0.69**		−0.72**	−0.22	0.80**	−0.28	−0.83**	−0.81**
LCC	−0.26**	−0.25 **	0.18**	−0.15**		0.12	−0.79**	0.46*	0.84**	0.65**
LNC	−0.03	0.01	0.35**	−0.08	0.24 **		0.08	−0.59**	0.01	0.40
LPC	−0.17**	−0.10	0.06	−0.03	0.16**	0.31**		−0.46*	−0.98**	−0.84**
LCN	−0.06	−0.10	−0.30**	0.04	0.07	−0.95**	−0.26**		0.49*	−0.02
LCP	0.10	0.06	−0.10	0.00	0.21**	−0.25**	−0.74**	0.32**		0.85**
LNP	0.08	0.08	0.31**	−0.06	0.14*	0.81**	−0.32**	−0.78**	0.21**	

*Note*: Abbreviations of traits are given in Table 3. Significant correlations are denoted with asterisks: “*” for *p* < 0.05, “**” for *p* < 0.01.

**TABLE 6:** Test of the phylogenetic signal of the leaf traits (Blomberg's *K* and *p*‐values).

Leaf traits	*K*	*p*
DOF	0.15	.29
LWC	0.31	**.01**
SLA	0.19	.16
TD	0.15	.27
LCC	0.07	.73
LNC	0.35	.09
LPC	0.02	.96
LCN	0.14	.33
LCP	0.02	.98
LNP	0.07	.62

*Note*: Bold indicates a significant phylogenetic signal. See Table 3 for abbreviations of the leaf traits.

## 4 DISCUSSION

### 4.1 Variation of leaf traits

Our results showed that interspecific variation was greater than intraspecific variation for most of the targeted leaf traits (Table 4). The linear mixed model and variance decomposition showed that the interspecific variation contributed more to the total variation than intraspecific in all leaf traits (Figure 2). Our results corroborate our first hypothesis that the variation in leaf traits of desert plants in northwest China is mainly from interspecific variation. In this study, the interspecific coefficient of variation (CV) of the plant leaf traits was 18.61%–122.13%, and the intraspecific CV was 8.05%–51.58%, this is consistent with most previous studies (de la Riva et al., 2016; Keddy, 1992; McGill et al., 2006; Shipley et al., 2016). Furthermore, the magnitude of leaf trait variation varies extensively with plant types and geographical distribution. This implies, plants converge or diverge with the habitat, which may be related to the plant adaptation strategies to their geographical environment (Chalmandrier et al., 2017; Levin, 1992; Wiens, 1989).

A meta‐analysis of global plants showed that intraspecific variation accounts for an average of 25% of total trait variation within the community and 32% among communities (Siefert et al., 2015). If plant traits are affected by extreme environments (Fajardo & Piper, 2011), variation in traits increases in areas with low species richness (Albert et al., 2011; Messier et al., 2016) and on gradients (Siefert et al., 2015). In this study, intraspecific variation was greater than or close to the interspecific variation for a few leaf traits, for example, TD for shrubs and SLA for herbs. This hints that intraspecific variation is indeed a source of leaf trait variation and cannot be ignored. Additionally, our study material was from distinct habitats (Table 1) and the external environment also drove a certain level of intraspecific variation since plants evolved to cope with local adversity (Westoby & Wright, 2006). If the intraspecific variation is overlooked and only traits at the interspecific level are considered, the magnitude of overlap of niches and traits among species will be severely underestimated (Albert, 2015; Albert et al., 2010), and thus the relative role of species in the competition. On the contrary, when the intraspecific variation is considered, it can reflect phenotypic plasticity due to individual genotype variation and habitat heterogeneity (Auger & Shipley, 2013; Fajardo & Siefert, 2016). Quantifying intraspecific variation could therefore reveal neglected community patterns (Jung et al., 2010).

In future trait‐based studies, we should not simply use species‐level trait averages instead of individual‐level data and ignore intraspecific variation but should study plant adaptation strategies to the environment based on individual‐level sampling. Integrating intraspecific and interspecific variation of traits will better reveal the mechanism of plant community assembly and biodiversity maintenance (Jiang et al., 2018, 2021).

### 4.2 Correlations among leaf traits and functions

Our results suggest that the correlation in leaf traits of shrubs aligned with the LES theory which confirms our hypothesis for the desert shrubs. The correlations between SLA and LNC, TD and LNC and SLA, SLA and LPC, LNC and LPC were consistent with the LES predictions (Figure 4a–d, f). This shows a trade‐off between acquisitive and conservative strategies in shrubs. Additionally, SLA and LNC were the principal axes of leaf trait variation in shrubs (Figure 3b), and some shrub species also had higher LNC and SLA (Figure 3a). This may be due to the increased nitrogen utilization in photosynthesis (Zhang et al., 2018). Meanwhile, shrub species may alleviate the effect of mutual leaf shading on photosynthesis (Yang et al., 2010) and accelerate the rate of gas exchange. This increased the efficiency of nitrogen utilization, accelerated the photosynthetic rate, and improved the water use efficiency (Navarro & Hidalgo‐Triana, 2021). These results are consistent with other dryland shrubs. For example, Puglielli and Varone (2018) found that the winter leaves of rockrose shrubs in the Mediterranean exhibited a fast acquisitive resource strategy. These results suggest that shrubs may adopt a rapid resource acquisition strategy. However, the remaining shrubs had smaller LNC and SLA (Figure 3a). This discrepancy might be explained by interspecific variations of leaf functional traits in shrubs (Li et al., 2022; Wang et al., 2020).

Contrary to our hypothesis for herbs, intercorrelation in leaf traits did not corroborate the LES in herbaceous plants. According to the LES, LNC and LPC should be positively correlated. However, we observed a negative relationship in studied herbs (Figure 4f). This negative correlation may be driven by legume species, for example, *Sophora alopecuroides*. Previous studies have shown that *Sophora alopecuroides* leaves had higher LNC and lower LPC (Cui et al., 2018). Moreover, we found no correlation between TD and LNC, LPC and TD in the herbs (Figure 4b, e), suggesting no trade‐offs between herbs' nitrogen and phosphorus acquisitive and conservative strategies. Therefore, desert herbs may not conform to the LES hypothesis.

In this study, the soil water and nutrient conditions differed at each of the four sites. However, PCA results (Figure 3b, c) demonstrate that environmental variations did not affect desert shrubs and herbs' resource strategy. Several studies have reported that resource availability has a limited impact on LES (Chen et al., 2020; Liu et al., 2010; Wright et al., 2004). For example, Wright et al. (2004) found that LES under different climatic gradients had the same trade‐off relationship. Yet, a study in subtropical China showed that woody species in high‐light environments had higher nutrient, photosynthetic, and resource acquisition capacity compared with those in low‐light environments (Zhao et al., 2017). They argue that this result may be due to light‐limitation‐induced interspecific and intraspecific variation in woody plants in subtropical regions (Zhao et al., 2017).

**FIGURE 5:**
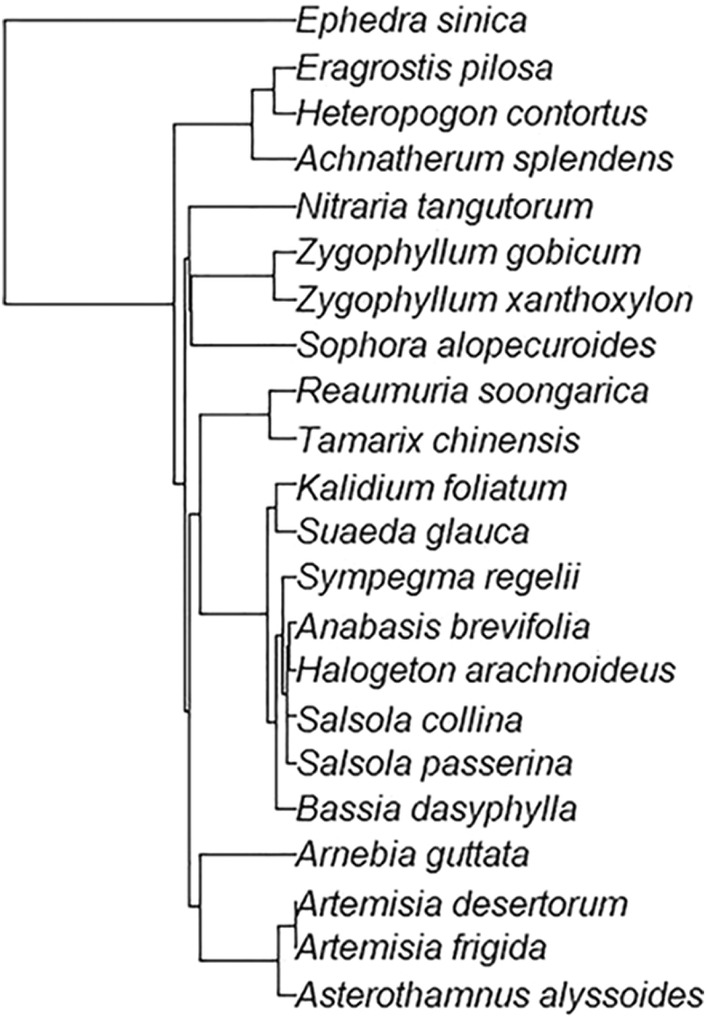
Phylogenetic relationships of 22 species of desert plants.

Leaf traits are in a state of dynamic change, but trade‐offs between different traits are relatively stable ecological strategies that plants have developed during evolution (Eviner & Chapin III, 2003; Krishna et al., 2021). Phylogenetics shows the entire evolution of species from origin to evolution (Figure 5). In this study, only the leaf water content had a significant phylogenetic signal, and the K values of other traits were relatively small (Table 6). This suggests that the evolution of leaf traits of these 22 desert plants tends to be more random (Ding et al., 2012; Swenson, 2013). The correlation between leaf traits changed under phylogenetic independent contrasts (PIC). Some traits (e.g., TD and DOF, LNC and LPC, and LNC and SLA) were significantly correlated in Pearson's correlation but not correlated after PIC. This indicates that these leaf traits are phylogenetically conserved. On the other hand, some leaf traits (SLA and TD, SLA and LNP) significantly correlated in both Pearson's correlation and after PIC, suggesting that these correlations were independent of phylogeny. These results indicate that trade‐offs between leaf traits are influenced by phylogeny in the studied 22 desert plants.

## 5 CONCLUSIONS

Interspecific variation in leaf traits of 22 desert plants in northwest China was greater than those of intraspecific variation. However, intraspecific variation still contributed to a certain amount of the total variation and thus should not be overlooked. There were trade‐offs between leaf traits in desert plants, which were mediated by phylogeny. SLA and LNC, TD and LNC, TD and SLA, LPC and SLA, LNC and LPC were correlated in shrubs, and these relationships are consistent with that assumed by the LES. Contrarily, we did not find a generalized trade‐off between LES traits in herbaceous plants studied here. Therefore, the shrubs support the LES hypothesis, but the herbs do not.

## AUTHOR CONTRIBUTIONS

**Hongyong Wang:** Conceptualization (lead); formal analysis (lead); methodology (equal); writing – original draft (lead); writing – review and editing (equal). **Jie Yang:** Investigation (lead); resources (equal); supervision (equal). **Tingitng Xie:** Funding acquisition (lead); resources (lead). **Li Ma:** Data curation (lead). **Furong Niu:** Writing – review and editing (equal). **Cai He:** Resources (equal). **Lishan Shan:** Conceptualization (equal); funding acquisition (lead); methodology (lead); writing – review and editing (lead).

## CONFLICT OF INTEREST STATEMENT

The authors declare no conflict of interest.

## Data Availability

The data supporting the results are available in a public repository at: https://doi.org/10.5061/dryad.jm63xsjf7.
